# RNA Dysregulation in Amyotrophic Lateral Sclerosis

**DOI:** 10.3389/fgene.2018.00712

**Published:** 2019-01-22

**Authors:** Zoe Butti, Shunmoogum A. Patten

**Affiliations:** INRS-Institut Armand-Frappier, National Institute of Scientific Research, Laval, QC, Canada

**Keywords:** ALS (amyotrophic lateral sclerosis), FUS, C9orf72, TDP-43, RNA processing, RNAi (RNA interference), antisense oligonucleotide-drug conjugates

## Abstract

Amyotrophic lateral sclerosis (ALS) is the most common adult-onset motor neuron disease and is characterized by the degeneration of upper and lower motor neurons. It has become increasingly clear that RNA dysregulation is a key contributor to ALS pathogenesis. The major ALS genes *SOD1, TARDBP, FUS*, and *C9orf72* are involved in aspects of RNA metabolism processes such as mRNA transcription, alternative splicing, RNA transport, mRNA stabilization, and miRNA biogenesis. In this review, we highlight the current understanding of RNA dysregulation in ALS pathogenesis involving these major ALS genes and discuss the potential of therapeutic strategies targeting disease RNAs for treating ALS.

## Introduction

Amyotrophic lateral sclerosis (ALS) is a progressive and fatal neurodegenerative disorder of motor function. It is characterized by the selective degeneration of the lower and upper motor neurons. Among the symptoms of this disease are progressive muscle weakness and paralysis, swallowing difficulties and breathing impairment due to respiratory muscle weakness that ultimately causes death, usually within 2–5 years following clinical diagnosis (Kiernan et al., 2011). Though most cases of ALS are sporadic, some families (10%) demonstrate a clinically indistinguishable form of ALS with clear Mendelian inheritance and high penetrance (Pasinelli and Brown, 2006). Treatments to slow the progression of ALS to date remains riluzole (Bensimon et al., 1994) and edaravone (Abe et al., 2014) but they are only modestly effective. However, in the past couple years, there has been a real encouragement in witnessing potentially efficacious treatments, such as Masitinib and Pimozide (Trias et al., 2016; Patten et al., 2017; Petrov et al., 2017) claiming to demonstrate clinical benefit. Furthermore, RNA-targeted therapies are currently intensively being evaluated as potential strategies for treating this ALS (Schoch and Miller, 2017; Mathis and Le Masson, 2018). There is indeed hope to have new and potentially more effective treatment options available for ALS in the near future.

Mutations in over more than 20 genes contribute to the etiology of ALS (Chia et al., 2018) (Table 1). Amongst these genes, the major established causal ALS genes are *SOD1* (Cu-Zn superoxide dismutase 1), *TARDBP* (transactive response DNA Binding protein 43kDa), *FUS* (fused in sarcoma) and hexanucleotide expansion repeat in Chromosome 9 Open Reading Frame 72 (*C9ORF72*). These genetic discoveries have led to the development of animal models (Julien and Kriz, 2006; Kabashi et al., 2010; Patten et al., 2014; Picher-Martel et al., 2016) that permitted the identification of key pathobiological insights. Currently, RNA dysregulation appears to be a major contributor to ALS pathogenesis. Indeed, TDP-43 and FUS are deeply involved in RNA processing such as transcription, alternative splicing and microRNA (miRNA) biogenesis (Buratti et al., 2004, 2010; Polymenidou et al., 2012). Mutations in *C9ORF72*, lead to a toxic mRNA gain of function through RNA foci formation, and the subsequent sequestration in stress granules and altered activity of RNA-binding proteins (Barker et al., 2017). In addition to the major ALS genes, other ALS genes including ataxin-2 (*ATXN2*) (Ostrowski et al., 2017), TATA-box binding protein associated factor 15 (*TAF15*) (Ibrahim et al., 2013), heterogeneous nuclear ribonucleoprotein A1 (*hnRNPA1*) (Dreyfuss et al., 1993), heterogeneous nuclear ribonucleoprotein A2 B1 (*hnRNPA2 B1*) (Alarcon et al., 2015), matrin 3 (*MATR3*) (Coelho et al., 2015), Ewing’s sarcoma breakpoint region 1 (*EWSR1*) (Duggimpudi et al., 2015), T-cell-restricted intracellular antigen-1 (*TIA1*) (Forch et al., 2000), senataxin (*SETX*) and angiogenin (*ANG*) (Yamasaki et al., 2009), play critical role in RNA processing (Table 1).

**Table 1:** ALS genes and their involvement in RNA processing.

Gene	Protein encoded	Regulation of RNA processing
*SOD1*	Superoxide dismutase 1	Yes
*TARDBP*	Tar-DNA-binding protein-43	Yes
*FUS*	Fused in sarcoma	Yes
*C9orf72*	C9orf72	Yes
*ATXN2*	Ataxin-2	Yes
*TAF15*	TATA-box binding protein associated factor 15	Yes
*UBQLN2*	Ubiquilin 2	No
*OPTN*	Optineurin	No
*KIF5A*	Kinesin family member 5A	No
*hnRNPA1*	Heterogeneous nuclear ribonucleoprotein A1	Yes
*hnRNPA2 B1*	Heterogeneous nuclear ribonucleoprotein A2/B1	Yes
*MATR3*	Matrin 3	Yes
*CHCHD10*	Coiled-coil-helix-coiled-coil-helix domain containing 10	No
*EWSR1*	EWS RNA binding protein 1	Yes
*TIA1*	TIA1 cytotoxic granule associated RNA binding protein	Yes
*SETX*	Senataxin	Yes
*ANG*	Angiogenin	Yes
*CCNF*	Cyclin F	No
*NEK1*	NIMA related kinase 1	No
*TBK1*	TANK binding kinase 1	No
*VCP*	Valosin containing protein	No
*SQSTM1*	Sequestosome 1	No
*PFN1*	Profilin 1	No
*TUBB4A*	Tubulin beta 4A class IVa	No
*CHMP2B*	Charged multivesicular body protein 2B	No
*SPG11*	Spatacsin vesicle trafficking associated	No
*ALS2*	Alsin Rho guanine nucleotide exchange factor	No

**FIGURE 1:**
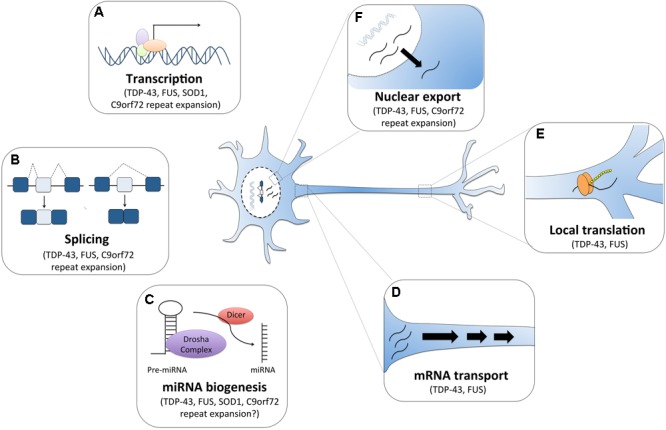
RNA dysfunction in amyotrophic lateral sclerosis (ALS). Major ALS mutations may disrupt RNA processing by several mechanisms. For instance, **(A)** mutations in ALS genes *SOD1, TDP-43, FUS* and *C9orf72* can alter gene expression. **(B)** The RNA binding proteins TDP-43 and FUS can affect global splicing machinery. Dipeptide repeat proteins from *C9orf72* intronic expansion can also alter splicing patterns of specific RNAs. **(C)** TDP-43, FUS, and dipeptide proteins can also promote microRNA biogenesis as components of the Drosha and Dicer complexes. TDP-43 and FUS also alter mRNA transport **(D)** and local translation **(E)**. **(F)** TDP-43 and FUS predominantly reside in the nucleus, but when mutated they are can mislocalization to the cytoplasm where they bind and regulate different sets of RNAs including the export and mislocalization of other transcripts to the cytoplasm. Poly-PR dipeptide can also bind nuclear pores channels blocking the import and export of molecules.

In this review, we focus on the four major ALS-associated genes (*SOD1, TARDBP, FUS*, and *C9orf72*) and present how they play critical roles in various RNA pathways. We particularly highlight recent developments on the dysregulation of RNA pathways (Figure 1) as a major contributor to ALS pathogenesis and discuss the potential of RNA-targeted therapies for ALS.

## Tar DNA Binding Protein (TDP-43)

A major advance in our understanding of cellular mechanisms in ALS came from the identification of causative mutations in the *TARDBP* gene (Kabashi et al., 2008; Sreedharan et al., 2008). This gene encodes for the evolutionarily conserved RNA/DNA binding protein, TDP-43. It is a protein that is normally nuclear, however, in cases of *TARDBP* mutations, it is mislocalized to the cytoplasm and forms aggregates (Van Deerlin et al., 2008; Winton et al., 2008b). It is found in the pathological aggregates in motor neurons in the majority of cases of ALS (Neumann et al., 2006). It is believed that TDP-43 aggregation leads to a gain of toxicity and its nuclear depletion results to a loss of function of TDP-43. Indeed, several studies have demonstrated that either overexpression or knockdown of TDP-43 causes neurodegeneration and ALS phenotypes (Kabashi et al., 2010; Stallings et al., 2010; Iguchi et al., 2013; Yang et al., 2014). For instance, the expression of the mutant TDP-43^A315T^ in the *C. elegans*’ GABAergic motor neurons results in age-dependent motility defects and neurodegeneration (Vaccaro et al., 2012). In drosophila, overexpression of TDP-43 in motor neurons was found to cause cytoplasmic accumulation of TDP-43 aggregates, neuromuscular junction (NMJ) morphological defects and cell death (Li et al., 2010). Similarly, the loss of TDP-43 reduced locomotion and lifespan (Feiguin et al., 2009; Diaper et al., 2013). Implications of TDP-43 loss and toxic gain-of-function in impaired motility, neurodegeneration and survival were further confirmed in higher model systems such as the zebrafish (Kabashi et al., 2010) and mice (Wegorzewska et al., 2009; Iguchi et al., 2013). Altogether, these reports strongly suggest that alterations in the level of TDP-43 are detrimental to neuronal function and survival.

TDP-43 contains two RNA recognition motifs (RRM1-2), a glycine rich domain in the C-terminus and nuclear localization and export signals (NLS and NES) (Buratti and Baralle, 2001; Winton et al., 2008a). TDP-43 plays a major role in multiple steps of RNA processing such as splicing, RNA stability and mRNA transport (Buratti and Baralle, 2008). For instance, TDP43 has been shown to bind to mRNA and regulate the expression of other proteins implicated in ALS and other neurodegenerative diseases such as FUS, Tau, ATXN 2 and progranulin (Polymenidou et al., 2011; Sephton et al., 2011; Tollervey et al., 2011). This suggests that TDP-43 may be a central component in the pathogenesis of several neurodegenerative conditions (Polymenidou et al., 2011). By RNA-seq analysis, Polymenidou et al. (2011) reported that TDP-43 is required for regulating the expression of 239 mRNAs, many of those encoding synaptic proteins. Several independent studies have corroborated that TDP-43 plays an important role in regulating genes involved in synaptic formation and function and in the regulation of neurotransmitter processes (Godena et al., 2011; Sephton et al., 2011; Colombrita et al., 2012; Narayanan et al., 2013; Chang et al., 2014). Examples of such genes are neurexin (*NRXN1-3*) (Polymenidou et al., 2011), neuroligin (*NLGN1-2*) (Polymenidou et al., 2011), scaffolding protein Homer2 (Sephton et al., 2011), microtubule-associated protein 1B (*MAP1B*) (Coyne et al., 2014), GABA receptors subunits (*GABRA2, GABRA3*) (Narayanan et al., 2013), AMPA receptor subunits (*GRIA3, GRIA4*) (Sephton et al., 2011; Narayanan et al., 2013), syntaxin 1B (Narayanan et al., 2013), and calcium channel cacophony (Chang et al., 2014). The development of TDP-43 animal models has offered the opportunity to explore synaptic alterations in ALS (Feiguin et al., 2009; Armstrong and Drapeau, 2013; Handley et al., 2017) and continuous efforts are being made to identify compounds that can facilitate synaptic transmission in ALS (Patten et al., 2017). Armstrong and Drapeau (2013) reported that expression of mutant *TARDP^G348C^* mRNA in zebrafish resulted in impaired synaptic transmission, reduced frequency of miniature endplate currents (mEPCs) and reduced quantal transmission. Remarkably, they also demonstrated that all these synaptic dysfunction features in their zebrafish *TARDBP* mutant were stabilized by chronic treatment the L-type calcium channel agonists (Armstrong and Drapeau, 2013). In drosophila neurons, TDP-43 depletion was shown to reduce dendritic branching as well as synaptic formation (Feiguin et al., 2009; Lu Y. et al., 2009). Overexpression or knocking down TDP-43 in cultured mammalian neurons also led to reduced dendritic branching (Herzog et al., 2017). In TDP-43*^A315T^* mice, Handley et al. (2017) showed that expression of mutant TDP-43 alters dendritic spine development, spine morphology and neuronal synaptic transmission. Collectively, these independent studies on several model systems, suggest that TDP-43 may play an important role in neuronal morphology, synaptic transmission and neuronal plasticity likely via regulation of RNA processing of various synaptic genes (Godena et al., 2011; Sephton et al., 2011; Colombrita et al., 2012; Narayanan et al., 2013; Chang et al., 2014).

TDP-43 is also known to act as a splicing regulator to reduce its own expression level by binding to the 3′ UTR of its own pre-mRNA (Ayala et al., 2011). Additionally, it functions as a splicing factor whose depletion or overexpression can affect the alternative splicing of specific targets (Polymenidou et al., 2011; Tollervey et al., 2011). Indeed, the alternative splicing of several genes were reported to be altered in human CNS tissues from TDP-43 ALS cases (Shiga et al., 2012; Yang et al., 2014). For instance, the level of the polymerase delta interacting protein 3 (*POLDIP3*) variant-2 mRNA (lacking exon 3) was significantly increased in the CNS of ALS patients with ALS, while that of variant-1 mRNA remained unchanged (Shiga et al., 2012). This was consistent with findings that TDP-43 directly regulates the inclusion of exon 3 of *POLDIP3* and that depletion of TDP-43 in cell culture models increased variant-2 mRNA (Shiga et al., 2012). TDP-43 has also been shown to regulate splicing of the cystic fibrosis transmembrane regulator (*CFTR*) gene and controls exon skipping by within the pre-mRNA (Buratti et al., 2004). Importantly, it controls the alternative splicing of apolipoprotein AII (*APOAII*) (Mercado et al., 2005) and survival of motor neuron (*SMN)* transcripts (Bose et al., 2008). Specifically, TDP-43 was shown to enhance the inclusion of exon 7 during the maturation of human S*MN2* pre-mRNA, which results to an increase in full-length *SMN2* mRNA level in neurons (Bose et al., 2008). Furthermore, recently TDP-43 was shown to bind to *HNRNPA1* pre-mRNA to modulate its alternative splicing (Deshaies et al., 2018). TDP-43 depletion resulted in exon7B inclusion, culminating in a longer hnRNAP A1B isoform that is aggregation-prone and cytotoxic (Deshaies et al., 2018). Collectively, these studies demonstrated that loss of TDP-43 results to alterations in alternative splicing of many genes and some of which, for example *HNRNPA1*, can contribute to cellular vulnerability. It would be interesting further to investigate the contribution of the alteration of splicing of these genes (*POLDIP3, CFTR, APOAII, SMN2, HNRNPA1*) to the pathogenesis of ALS.

TDP-43 is actively transported along axons and co-localizes with other well-known transport RNA binding proteins close to synaptic terminals (Wang I.F. et al., 2008; Narayanan et al., 2013). It was reported that TDP-43 mutations impair mRNA transport function *in vivo* and *in vitro* (Alami et al., 2014). In addition to a role in mRNA transport, TDP-43 also acts as a regulator of mRNA stability (Strong et al., 2007; Fiesel and Kahle, 2011). It was shown to directly interacts with the 3′ UTR of neurofilament light chain (*NFL)* mRNA to stabilize it (Strong et al., 2007) and associates with *futsch/MAP1B* mRNA in Drosophila to regulates its localization and translation (Coyne et al., 2014). Particularly, TDP-43 was found to interact with 14-3-3 protein subunits to modulate the stability of the *NFL* mRNA (Volkening et al., 2009). Abnormal regulation of *NFL* mRNA has been observed in ALS patients (Wong et al., 2000) and disruption of *NFL* mRNA stoichiometry leads to motor neuron death and symptoms of ALS in animal models (Xu et al., 1993; Julien et al., 1995). It is, thus, very likely that TDP-43 mutations may cause motor neuron degeneration by interfering with RNA processing of *NFL* mRNA.

Other important identified targets regulated by TDP-43 at mRNA level that may play a role in disease are *G3BP* (McDonald et al., 2011) and *TBC1D1* (Stallings et al., 2013). G3BP is an essential component of stress granules, which are cytoplasmic non-membrane organelles that store translationally arrested mRNAs that accumulate during cellular stress (Kedersha and Anderson, 2007). Stress granules consists of polyadenylated mRNAs, translation initiation factors (e.g., eIF3, eIF4E, and eIF4G), small ribosomal subunits and a numerous RNA-binding proteins (Protter and Parker, 2016). TDP-43 is recruited to stress granules in cellular models upon exposure to different stressors (Colombrita et al., 2009; Liu-Yesucevitz et al., 2010; Bentmann et al., 2012). Importantly, cytosolic TDP-43 mutants are more efficiently recruited to stress granules upon cellular stress compared to nuclear wild-type TDP-43 (Liu-Yesucevitz et al., 2010). Prolonged stress is thought to promote sequestration of TDP-43 and their mRNA targets in stress granules; thereby inhibiting translation and potentially contributing to ALS progression (Ramaswami et al., 2013).

## Fused in Sarcoma (FUS)

Mutations in *FUS* are detected in 4–5% of familial ALS patients as well as in sporadic ALS (Kwiatkowski et al., 2009; Vance et al., 2009; Corrado et al., 2010; DeJesus-Hernandez et al., 2010). FUS is an RNA/DNA-binding protein of 526 amino acids, consisting of an RNA-recognition motif, a SYGQ (serine, tyrosine, glycine and glutamine)-rich region, several RGG (arginine, glycine and glycine)-repeat regions, a C2C2 zinc finger motif and a nuclear localization signal (NLS) (Iko et al., 2004). C-terminal ALS FUS mutations disrupt the NLS region and the nuclear import of FUS; resulting in cytoplasmic accumulation (Kwiatkowski et al., 2009; Vance et al., 2009).

Similarly to TDP-43, FUS plays multiple roles in RNA processing by directly binding to RNA. Using CLIP-based methods, several groups have identified thousands of RNA targets bound by FUS in various cell lines (Hoell et al., 2011; Colombrita et al., 2012; Ishigaki et al., 2012), and brain tissues (Lagier-Tourenne et al., 2012; Rogelj et al., 2012). Interestingly, FUS was identified in spliceosomal complexes (Rappsilber et al., 2002; Zhou et al., 2002) and interacting with several key splicing factors (such as hnRNP A1, YB-1) (Rapp et al., 2002; Meissner et al., 2003; Kamelgarn et al., 2016) as well as with the U1 snRNP (Yamazaki et al., 2012; Yu et al., 2015). FUS regulates splicing events for neuronal maintenance and survival (Lagier-Tourenne et al., 2012). Given that FUS plays an essential role in splicing regulation, the consequence of its loss of function in ALS on RNA splicing has been immensely investigated (Lagier-Tourenne et al., 2012; Zhou Y. et al., 2013; Reber et al., 2016). For instance, Reber et al. (2016) showed by mass spectrometric analysis that minor spliceosome components are highly enriched among the FUS-interacting proteins. They further reported that FUS interacts with the minor spliceosome and directly regulates the removal of minor introns (Reber et al., 2016). Moreover, the FUS^P525L^ ALS mutation, which destroys the NLS and results in cytoplasmic retention of FUS (Dormann et al., 2010), inhibits splicing of minor introns and causes mislocalization of the minor spliceosome components U11 and U12 snRNA to the cytoplasm and inhibits splicing of minor introns (Reber et al., 2016). Loss of function of FUS led to splicing changes in more than 300 genes mice brains (Lagier-Tourenne et al., 2012) and importantly a vast majority minor intron containing mRNAs was altered (Reber et al., 2016). Corroborating the results with mouse brain, many minor intron-containing genes were found to be downregulated in FUS-depleted SH-SY5Y cells (Reber et al., 2016). FUS depletion has been shown to affect minor intron containing genes that are important for neurogenesis (*PPP2R2C*), dendritic development (*ACTL6B*) and action potential transmission in skeletal muscles (*SCN8A* and *SCN4A*) (Reber et al., 2016) and may contribute to ALS pathogenesis. FUS has also been shown to regulate alternative splicing of genes related to cytoskeletal organization, axonal growth and guidance such as the microtubule-associated protein tau (*MAPT)* (Ishigaki et al., 2012; Orozco et al., 2012; Rogelj et al., 2012), Netrin G1 (*NTNG1)* (Rogelj et al., 2012), neuronal cell adhesion molecule *(NRCAM)* (Rogelj et al., 2012; Nakaya et al., 2013) and the actin-binding *LIM* (*ABLIM1)* (Nakaya et al., 2013). For example, FUS knockdown has been shown to promote inclusion of exon 10 in the MAPT/tau protein and to significantly cause shortened axon length and growth cone enlargement (Orozco et al., 2012). Loss of function of FUS altered MAPT/tau isoform expression and likely disturbed cytoskeletal function impairing axonal growth and maintenance. Interestingly, axon retraction and denervation are early events in ALS (Boillee et al., 2006; Nijssen et al., 2017). Disruption of cytoskeleton function may thus play an important role in neurodegeneration in ALS.

Besides its functions in splicing, FUS has been proposed to regulate transcription by RNA polymerase II (RNAP2), RNA polymerase III (RNAP3) or cyclin D1 (Wang X. et al., 2008; Tan and Manley, 2010; Brooke et al., 2011; Schwartz et al., 2012; Tan et al., 2012). For instance, transcriptomic analyses showed that knockdown of FUS results in differential expression several genes (Lagier-Tourenne et al., 2012; Nakaya et al., 2013) including many mRNAs encoding proteins important for neuronal function. Transcriptome changes have also been observed in human motoneurons obtained from FUS mutant induced pluripotent stem cells (IPSCs) (De Santis et al., 2017) and transgenic FUS knockin mice (Scekic-Zahirovic et al., 2016). Alterations in the expression of several genes involved in pathways related to cell adhesion, apoptosis, synaptogenesis and other neurodegenerative diseases were reported in these FUS models (Fujioka et al., 2013; Scekic-Zahirovic et al., 2016; De Santis et al., 2017). Among these genes *TAF15*, which is mutated in some case of ALS (Couthouis et al., 2011), has been found to be upregulated in several ALS FUS models including human mutant IPSC derived motoneurons (De Santis et al., 2017), FUS knockout and knockin mouse (Kino et al., 2015; Scekic-Zahirovic et al., 2016). However, it remains to be determined whether *TAF15* upregulation upon FUS loss- or toxic gain- of function contributes to ALS pathogenesis.

FUS is also incorporated into stress granules under cellular stress conditions (Sama et al., 2013). Sequestration of FUS and its protein partners into these cytoplasmic organelles appears to contribute to ALS pathogenesis (Yasuda et al., 2013). An example of such a protein partner is Pur-alpha, which co-localizes with mutant FUS and becomes trapped in stress granules in stress conditions, as reported in ALS patient cells carrying FUS mutations (Di Salvio et al., 2015; Daigle et al., 2016). It has been shown that FUS physically interacts with Pur-alpha. *In vivo* expression of Pur-alpha in Drosophila significantly exacerbates the neurodegeneration caused by mutated FUS. Conversely, Di Salvio et al. (2015) showed that the downregulation of Pur-alpha in neurons expressing mutated FUS significantly improves fly climbing activity. It was subsequently demonstrated that overexpression Pur-alpha inhibits cytoplasmic mislocalization of mutant FUS and promotes neuroprotection (Daigle et al., 2016). However, the function of Pur-alpha in regulating ALS pathogenesis remains elusive.

## Superoxide Dismutase-1 (SOD1)

Unlike TDP43 and FUS, SOD1 does not contain RNA-binding motifs, however, several reports have demonstrated a potential role of mutant SOD1 in regulating RNA metabolism (Menzies et al., 2002; Lu et al., 2007; Lu L. et al., 2009; Chen et al., 2014). Particularly, mutant SOD1 can bind mRNA species such as vascular endothelial growth factor (*VEGF*) and *NFL* and negatively affects their expression, stabilization and function (Menzies et al., 2002; Lu et al., 2007; Lu L. et al., 2009; Chen et al., 2014). More precisely, mutant SOD1 can directly bind to specific adenylate- and uridylate-rich stability elements (AREs) located in the 3′ UTR of transcripts of *VEGF* (Lu et al., 2007) and *NFL* (Chen et al., 2014). It is believed that such a gain of abnormal protein–RNA interactions can be caused by SOD1 misfolding that results in the exposure of polypeptide portions with the ability to bind nucleic acids (Kenan et al., 1991; Tiwari et al., 2005).

Binding of mutant SOD1 to the 3′ UTR of the *VEGF* mRNA results in the sequestration of other ribonucleoproteins such as TIAR and HuR into insoluble aggregates. These interactions, which are specific to mutant SOD1, result in decline levels of *VEGF* mRNA, impairment of HuR function and ultimately hampering their neuroprotective actions during stress responses (Lu et al., 2007; Lu L. et al., 2009).

In motor neuron-like NSC34 cell lines expressing mutant SOD1 (G37R or G93A), the level of *NFL* mRNA is significantly reduced (Menzies et al., 2002). Reduction in *NFL* mRNA levels has also been reported in G93A transgenic mice and human spinal motor neurons from SOD1-ALS cases (Menzies et al., 2002). It is proposed that destabilization *NFL* mRNA by mutant SOD1, result to altered stoichiometry of neurofilament (NF) subunits and subsequent NF aggregation in motor neurons (Chen et al., 2014). NF inclusion in the soma and proximal axons of spinal motor neurons is a hallmark of ALS pathology (Hirano et al., 1984). In IPSC-derived model of ALS, a reduction of *NFL* mRNA level has been reported to result in NF aggregation and neurite degeneration (Chen et al., 2014). Altogether, these studies support a pathogenic role for dysregulation of RNA processing in SOD1-related ALS.

Interestingly, SOD1 has been shown to interact with TDP-43 to modulate *NFL* mRNA stability (Volkening et al., 2009). As mentioned above, TDP-43 was found to directly interact with the 3′ UTR of *NFL* mRNA to stabilize it (Strong et al., 2007). Altogether, these studies suggest that SOD1 and TDP-43 may act in a possible common action in regulating specific RNA stability. In the case of *NFL* mRNA, it would be interesting to investigate whether mutant SOD1 dislodges TDP-43 from the *NFL* mRNA in a manner that would affect its mRNA metabolism and potentially making NF prone to form aggregates.

Furthermore, there have been several transcriptome investigations in SOD1 human samples (D’Erchia et al., 2017), motor neuron-like NSC34 cell culture model (Kirby et al., 2005) and transgenic animals including mice (Lincecum et al., 2010; Bandyopadhyay et al., 2013; Sun et al., 2015), rat (Hedlund et al., 2010) and drosophila (Kumimoto et al., 2013). These studies have reported dysregulation of genes involved in pathways related to the neuroinflammatory and immune response, oxidative stress, mitochondria, lipid metabolism, synapse and neurodevelopment (Hedlund et al., 2010; Lincecum et al., 2010; Bandyopadhyay et al., 2013; Kumimoto et al., 2013; Sun et al., 2015; D’Erchia et al., 2017). However, in these studies it is not clear whether SOD1 directly or indirectly impact the regulation of the differentially expressed genes. In a recent elegant study, Rotem et al. (2017), compared transcriptome changes in SOD1 and TDP-43 models. They found that most genes that were altered in the SOD1^G93A^ model were not dysregulated in the TDP-43^A315T^ model, and vice versa (Rotem et al., 2017). There were, however, a few genes whose expressions were altered in both ALS models (Rotem et al., 2017). These findings are consistent with the ALS pathology, which is distinguishable between the ALS-related SOD1 phenotype and the TDP-43 phenotype. Although different cellular pathways are likely activated by SOD1 versus TDP-43, it is very plausible that they ultimately convergence onto common targets to result in similar motor neuron toxicity and ALS phenotype.

## C9orf72 Intronic Expansion

In 2011, a large GGGGCC hexanucleotide repeat expansion in the first intron or promoter region of the *C9orf72* gene has been discovered as a new cause of ALS (DeJesus-Hernandez et al., 2011; Renton et al., 2011). *C9orf72* repeat expansion mutations account for about 50% of familial ALS and 5–10% of sporadic ALS (Majounie et al., 2012). It remains a topic of debate whether the repeat expansion in *C9orf72* causes neurodegeneration primarily through a toxic gain of function, loss of function, or both. The *C9orf72* repeat expansion is transcribed in both the sense and antisense directions and leads to accumulations of repeat-containing RNA foci in patient tissues (Gendron et al., 2013). The formation of RNA foci facilitates the recruitment of RNA-binding proteins, causes their mislocalization and interferes with their normal functions (Simon-Sanchez et al., 2012; Donnelly et al., 2013; Lee et al., 2013; Gitler and Tsuiji, 2016). Indeed, RNA foci may bind RNA binding proteins and alter RNA metabolism (Donnelly et al., 2013; Lee et al., 2013; Mori et al., 2013a). For example, Mori et al. (2013a) and Hutvagner et al. (2001) showed that RNA foci can sequester hnRNP-A3 and repress its RNA processing function. Aborted transcripts containing the repeat can also disrupt nucleolar function (Haeusler et al., 2014). Importantly, these foci can sequester nuclear proteins such as TDP-43 and FUS, impacting expression of the their RNA targets and culminating in a range of RNA misprocessing events. Other RNA binding proteins binding to RNA foci include hnRNP A1, hnRNP-H, ADARB2, Pur-α, ASF/SF2, ALYREF and nucleolin (Donnelly et al., 2013; Lee et al., 2013; Sareen et al., 2013; Xu et al., 2013; Cooper-Knock et al., 2014; Haeusler et al., 2014). Antisense oligonucleotides (ASOs) targeting the C9orf72 repeat expansion suppress RNA foci formation, attenuate sequestration of specific RNA-binding proteins and reverse gene expression alterations in C9orf72 ALS motor neurons derived from IPSCs (Donnelly et al., 2013; Lagier-Tourenne et al., 2013).

Additionally, simple dipeptide repeats (poly-GA, poly-GP, poly-GR, poly-PA, and poly-PR) can be generated by repeat-associated non-ATG-dependent (RAN) translation of both the sense and antisense strands that have a variety of toxic effects (Ash et al., 2013; Mori et al., 2013b). Poly-PR and poly-GR can alter the splicing patterns of specific RNAs. For example, poly-PR has been shown to cause exon-skipping in *RAN* and *PTX3* RNA (Kwon et al., 2014). Dipeptides repeat proteins have also been found to be toxic by creating aggregates sequestrating cytoplasmic proteins (Freibaum and Taylor, 2017). Poly-GR dipeptide co-localizes with several ribosomal subunits and with a transcription factor elF3η (Zhang et al., 2018c). This suggests a ribosomal dysfunction, which implies a defect in RNA translation. In line with these findings, a recent report demonstrated that poly-PR co-localizes with the nucleolar protein, nucleophosmin, and reduces the expression of several ribosomal RNA (Suzuki et al., 2018). Suzuki et al. (2018) further showed that the reduction in the expression of ribosomal RNA results in neuronal cell death and this could be rescued by overexpression of an accelerator of ribosome biogenesis, Myc (Suzuki et al., 2018). RNA sequencing reveals that more than 6,000 genes are up or down regulated in mice that express the dipeptide construct in the brain (Zhang et al., 2018c). Other findings show that poly-PR dipeptide binds nuclear pores channels blocking the import and export of molecules. The dipeptide actually binds the nucleoporin proteins Nup54 and Nup98 that rim the central channel of the pore (Shi et al., 2017). The accumulation of poly-PR dipeptide at the nuclear pore was found to correlate with defect in nuclear transport of RNA and protein, which is consistent with previous findings (Freibaum et al., 2015; Zhang et al., 2015).

The last proposed mechanism involved in ALS pathogenesis is a haploinsufficiency due to the expansion of repetition leading to a decreased transcription of the gene and consequently to a decrease of its translation (Ciura et al., 2013). Studies have demonstrated that C9orf72 expansion repeat can interfere with transcription or splicing of *C9orf72* transcripts (Mori et al., 2013b; Haeusler et al., 2014; Highley et al., 2014). It has also been proposed that the C9orf72 expansion repeat could disrupt the C9orf72 promoter activity thereby reducing its expression (Gijselinck et al., 2016). Several studies have demonstrated alterations in the C9orf72 ALS transcriptome (Donnelly et al., 2013; Prudencio et al., 2015; Selvaraj et al., 2018). Interestingly, a recent article reported an increased expression of the calcium-permeable GluA1 AMPA receptor subunit in motoneurons derived from IPSC of patients with *C9orf72* mutations (Selvaraj et al., 2018). This alteration in AMPA receptor composition led to an enhanced motoneuron vulnerability to AMPA-induced excitotoxicity (Selvaraj et al., 2018). It remains to be determined whether the increased expression of GluA1 AMPA subunit is related to reduced levels of C9orf72, RNA foci and/or dipeptide repeats.

*C9orf72* has also been showed to be involved in the generation of stress granules (Maharjan et al., 2017) and sequestering other RNA binding proteins that are involved in nucleo-cytoplasmic transport (Zhang et al., 2015, 2018b). It has been found that stress granules observed in *C9orf72* mutants co-localizes with Ran GAP (Zhang et al., 2015, 2018b); which is known to activate Ran GTPase. This GTPase in involved in nucleo-cytoplasmic transport. It has also been published that expressing Ran GAP rescues the age-related motor defects in flies expressing the GGGGCC repeats (Zhang et al., 2018a). Very recently, it has also been reported that one of the dipeptide generated by the expansion has a role in formation of these stress granules (Zhang et al., 2018c). Moreover, importins and exportins are sequestered in stress granules; which also implies that protein transport in altered (Zhang et al., 2018b).

These toxic gain- or loss-of function mechanisms are thought to be all involved in synergy in ALS pathogenesis and it can be summed up that that altered RNA processing plays a key role in *C9orf72*-mediated toxicity through two ways. The first is altered processing of the expanded *C9orf72* transcript itself, in terms of altered transcription, splicing defects, nuclear aggregation and non-conventional translation (Barker et al., 2017). The second involves downstream and indirect changes in RNA processing of other transcripts. A thorough understanding of RNA metabolism dysregulation could definitely bring a major enlightenment on how *C9orf72* mutation leads to ALS and provide insights on therapeutic targets.

## Dysregulation of Microrna (miRNA) in ALS

Multiple mechanisms control the proper levels of RNA and subsequent protein expression; among these are microRNAs (miRNAs) (Catalanotto et al., 2016). They are endogenous small non-coding RNAs (approximately 22 nucleotides in length) that are initially transcribed by the RNA polymerase II as primary miRNA (pri-miRNAs) transcripts. These pri-miRNAs are processed into precursor miRNAs (pre-miRNAs) by the nuclear ribonuclease III (RNase III), DROSHA, and the double-stranded RNA-binding protein, DGCR8, which anchors DROSHA to the pri-miRNA transcript (Lee et al., 2003; Denli et al., 2004). Pre-miRNA is then exported into the cytoplasm by exportin-5 (Yi et al., 2003), where it is processed into a mature miRNA by the DICER enzyme (Hutvagner et al., 2001; Ketting et al., 2001). The mature miRNA is then incorporated with a ribonucleoprotein (RNP) complex with argonaute (AGO) proteins to form the RNA-induced silencing complex (RISC) (Hammond et al., 2001; Schwarz et al., 2003; Kawamata and Tomari, 2010), which mediates inhibition of translation and/or mRNA degradation of targeted transcripts that are complementary to the miRNA (Hutvagner and Zamore, 2002; Yekta et al., 2004). The recognition of mRNAs by miRNAs occurs through base-pairing interactions within the 3′-untranslated region (UTR) of the targeted mRNAs. Besides their well-known gene silencing functions, miRNAs can also induce up-regulation of their targets (Vasudevan et al., 2007; Lin et al., 2011; Truesdell et al., 2012; Vasudevan, 2012).

MiRNAs play important roles in several biological processes such as cell proliferation (Chen et al., 2006), cell differentiation (Naguibneva et al., 2006), apoptosis (Matsushima et al., 2011), and patterning of the nervous system (Johnston and Hobert, 2003). Interestingly, several miRNAs have been particularly shown to be essential for motor neuron development and survival (see review, Haramati et al., 2010). For example, in developing chick, it was demonstrated that the activation of the miRNA miR9 is necessary to suppress the expression of the transcription factor *onecut1*, which in turn helps to drive differentiation of neural progenitor cells into spinal motor neurons (Luxenhofer et al., 2014). It is believed that several miRNAs work in concert to establish motor neuron identity. Indeed, in addition to miR9, other miRNAs such as miR-128 (Thiebes et al., 2015), miR-196 (Asli and Kessel, 2010), miR-375 (Bhinge et al., 2016) have been shown to play a role in motor neuron differentiation and localization. Loss of DICER function within progenitor cells results in aberrant motor neuron development while its loss in motor neuron leads to progressive motor neuron degeneration (Haramati et al., 2010; Chen and Wichterle, 2012). Furthermore, miRNAs are important players for NMJ function, synaptic plasticity and for maintaining cytoskeletal integrity (see review, Hawley et al., 2017).

The ALS genes, TDP-43 and FUS, were identified in a protein complex with RNAse III DORSHA and shown to play a role in miRNA biogenesis (Freibaum et al., 2010; Da Cruz and Cleveland, 2011). TDP-43, in particular was shown to associate with proteins involved in the cytoplasmic cleavage of pre-miRNA mediated by the DICER enzyme (Freibaum et al., 2010). It is thus to no surprise that dysregulation of miRNAs has been observed in ALS (Li et al., 2013; Zhang et al., 2013; Dini Modigliani et al., 2014; Eitan and Hornstein, 2016). Indeed, mutations in *TARDBP* result in differential expression of miRNAs – miR-9, miR-132, miR-143, and miR-558 (Kawahara and Mieda-Sato, 2012; Zhang et al., 2013). Interestingly, the expression of several of these miRNAs (miR-9, miR-132, miR-143) and including others (such as miR-125, miR-192) are altered upon FUS depletion (Morlando et al., 2012). MiR-9 expression is also found to be upregulation in mutant SOD1 mice (Zhou F. et al., 2013). These dysregulated miRNAs are essential for motor neuron development and maintenance (Otaegi et al., 2011; Luxenhofer et al., 2014), axonal growth (Dajas-Bailador et al., 2012; Kawahara and Mieda-Sato, 2012) and synaptic transmission (Edbauer et al., 2010; Sun et al., 2012). Thus, these miRNA alterations likely contribute to the pathological phenotype observed in ALS.

Additionally, depletion of TDP-43 in cell culture systems has also been shown to change the total miRNA expression profile (Buratti et al., 2010). A similar observation was recently observed in motoneurons progenitors derived from human ALS IPSCs (Rizzuti et al., 2018). Particularly, it was reported that 15 miRNAs were dysregulated including disease-relevant miR-34a and miR504, which are known to be, implicated synaptic vesicle regulation and cell survival (Rizzuti et al., 2018). Additionally, another important miRNA, namely microRNA-1825, was found to be downregulated in CNS of both sporadic and familial ALS patients (Helferich et al., 2018). Interestingly, reduced levels of microRNA-1825 was demonstrated to cause a translational upregulation of tubulin-folding cofactor b (*TBCB*) which consequently to depolymerization and degradation of tubulin alpha-4A (*TUBA4A*), which is encoded by a known ALS gene (Helferich et al., 2018).

In several repeats diseases such as myotonic dystrophy, fragile X tremor and ataxia syndrome, toxic RNA from expansion repeats cause widespread RNA splicing abnormalities, degeneration of affected tissues (Miller et al., 2000) and alter miRNA processing (Sellier et al., 2013). Since its discovery, C9orf72 GGGGCC expansion repeat was also questioned as a disruptor of miRNA processing. Recently, the DROSHA protein was found to be mislocalized in dipeptide repeat protein-aggregates in frontal cortex and cerebellum C9orf72 ALS/FTLD patients (Porta et al., 2015). An involvement of the miRNA pathway in motor neuron impairment in ALS is evident and further investigations on miRNAs dysregulation in ALS pathogenesis could eventually lead to the identification of therapeutic targets.

## RNA-Targeted Therapeutics for ALS

**FIGURE 2:**
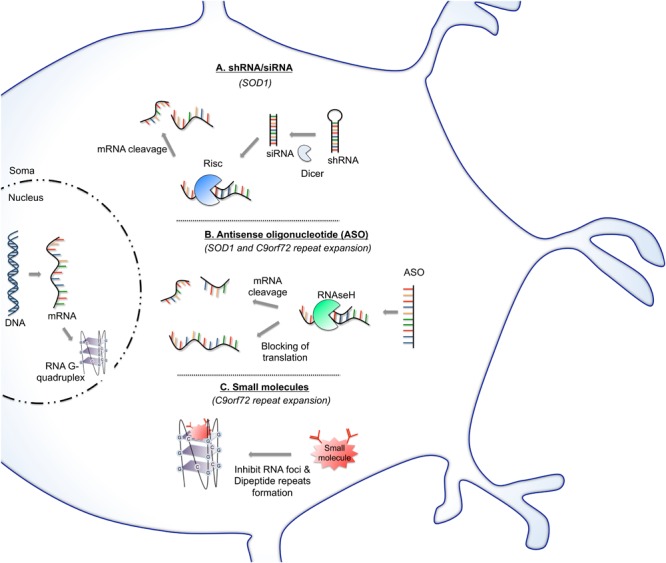
RNA-based therapy approaches for potentially treating ALS. (A) SiRNAs operate through RNA interference pathway. After strand unwinding, one siRNA strand binds argonaute proteins as part of the RNA-induced silencing complex (RISC) and is recruited to a target mRNA which is then cleaved. Virus can provide a means of shRNA, which will be cleaved once in the cytoplasm by dicer enzyme into siRNA. This approach has been evaluated to reduce the level of mutant SOD1 protein. (B) Antisense oligonucleotide (ASO) binds to targeted mRNA and induces its degradation by endogenous RNase H or blocks the mRNA translation. This strategy is being exploited as a potential therapeutic avenue in ALS aiming principally to reduce the protein level of SOD1 protein or by targeting of C9orf72 RNA foci. (C) Small molecules can be designed to target and stabilize RNA structures. This approach was particularly tested to stabilize G-quadruplex of C9orf72 GGGGCC repeat RNA. Stabilization of G-quadruplex structure reduces RNA foci formation and blocks repeat translation.

Our understanding of RNA biology has expanded tremendously over the past decades, resulting in new approaches to engage RNA as a therapeutic target. More precisely, RNA-targeted therapeutics have been developed to mediate the reduction or expression of a given target RNA by employing mechanisms such as RNA cleaving, modulation of RNA splicing, inhibition of mRNA translation into protein, inhibition of miRNA binding sites, increasing translation by targeting upstream open reading frames and disruption of RNA structures regulating RNA stability (Robertson et al., 2010; Fellmann and Lowe, 2014; Vickers and Crooke, 2014; Havens and Hastings, 2016; Liang et al., 2016). Therapeutics that directly target RNAs are promising for a broad spectrum of disorders, including the neurodegenerative diseases (Scoles and Pulst, 2018) and are currently under evaluation as potential strategies for treating ALS. The RNA therapeutics approaches include RNA interference (RNAi) and ASOs (Figure 2), both bind to their target nucleic acid via Watson-Crick base pairing and cause degradation of or inactivate the targeted mRNA (Burnett and Rossi, 2012). Recently, application of innovative drug discovery approaches has showed that targeting RNA with bioactive small molecules is achievable (Disney, 2013; Bernat and Disney, 2015). A few researchers including us are currently exploiting such a new type of RNA-targeted therapeutics to search for RNA-targeted small molecules as C9orf72 ALS therapeutics.

### RNA Interference (RNAi)

RNAi is an endogenous cellular mechanism to regulate mRNA. It operates sequence specifically and post-transcriptionally via the RISC (Carthew and Sontheimer, 2009). Methods of mediating the RNAi effects are via small interfering RNA (siRNA), short hairpin RNA (shRNA), and artificial miRNA (Fire et al., 1998; Moore et al., 2010; Chakraborty et al., 2017). These approaches can help to reduce the expression of mutant (toxic) gene and can provide significant therapeutic benefit in treating ALS and other neurodegenerative disease implicating aberrant accumulation of misfolded proteins.

The challenge of using siRNA for treating ALS is that it has to be designed to have the specificity and ability to reduce the aberrant mutant protein while leaving wild-type protein intact. Attempts were made to design siRNA, which could recognize just a single nucleotide alternation to selectively suppress mutant SOD1 (particularly G93A) expression leaving wild-type SOD1 intact (Yokota et al., 2004; Wang H. et al., 2008). The design of siRNA G93A.1 and G93A.2 by Yokota et al. (2004) were found to successfully suppress the expression of approximately 90% of mutant SOD1 G93A. Importantly, both siRNA had virtually little or no effect on wild-type SOD1 expression (Yokota et al., 2004). To achieve long-term expression of siRNA in cells, the use of viral delivery system has proved powerful to provide a continuous delivery and expression of shRNA in sufficient quantities (Bowers et al., 2011). Indeed, diverse viral vectors have been studied such as adeno-associated virus (AAV), lentivirus (LV), and rabies-glycoprotein-pseudotyped lentivirus (RGP-LV) (Raoul et al., 2005; Wu et al., 2009). Recombinant AAVs are currently the choice of RNAi treatment vehicle for neurological diseases because they are non-pathogenic and safe (Maguire et al., 2014; Smith and Agbandje-McKenna, 2018). Several studies have aimed at engineering AAV serotypes with better cell-type and tissue specificities and an improved immune-evasion potential (Gao et al., 2005; Weinmann and Grimm, 2017). AAV9 and AAVrh10 serotypes have been shown to cross the blood–brain barrier and efficiently transduce cells in the CNS, with widespread and sustained transgene expression in the spinal cord and brain even after just a single injection (Thomsen et al., 2014; Dirren et al., 2015; Borel et al., 2016). Importantly, they can efficiently target neurons and astrocytes, making them the most applicable delivery systems for treating ALS.

Several researchers have independently use siRNA or shRNA to silence mutant SOD1 expression *in vitro* and *in vivo* (Miller et al., 2005; Raoul et al., 2005; Ralph et al., 2005; Foust et al., 2013). Intramuscular delivery of siRNA targeting mutant *SOD1* in *SOD1^G93A^* mice delays the onset of motor neuron symptoms and extend their survival (Miller et al., 2005). Similarly, *SOD1^G93A^* mice treated with injection of AAV encoding shRNA against human *SOD1* mRNA (h*SOD1*) exhibited delayed diseases onset and significantly increased their survival by 23% (Foust et al., 2013). The same group later demonstrated the efficacy of this approach in *SOD1^G93A^* rats, showing that silencing of h*SOD1* expression selectively in the motor cortex also delayed disease onset and prolonged survival (Thomsen et al., 2014). Silencing of *SOD1* using an artificial miRNA (miR-*SOD1*) systemically delivered using the viral vector AAVrh10 in *SOD1^G93A^* mice was also found to significantly delayed disease onset, preserved muscle motor functions and extended survival (Borel et al., 2016). Interestingly, similar findings were observed in non-human primates treated with AAVrh10-miR-*SOD1* (Wang et al., 2014; Borel et al., 2016). These findings suggest that miRNA silencing strategy warrants further investigations and may offer promise for the development for the treatment of *SOD1*-related ALS.

### Antisense Oligonucleotides (ASOs)

The concept of ASOs was first introduced in 1978, when Stephenson and Zamecnik used a chemically modified oligonucleotide, designed to bind to its complementary sequence in a Rous sarcoma virus transcript to inhibit its gene expression and viral replication (Stephenson and Zamecnik, 1978). ASOs are synthetic single-stranded oligonucleotides that activate the RNAse H, an endonuclease in the nucleus, to degrade the complementary mRNA. They can be designed to specifically target mutant RNAs or mRNA splicing (Bennett and Swayze, 2010). An ASO therapy based (nusinersen) approach designed to promote exon skipping has proven to be very effective in treating spinal muscular atrophy (SMA) in clinical trials (Chiriboga et al., 2016; Finkel et al., 2016; Mendell et al., 2017; Scoto et al., 2017). In late 2016, this antisense drug (marketed as Spinraza) has received FDA approval for the treatment of SMA. This was the first exciting success of ASO therapeutics in neurodegeneration and a significant milestone for ASO therapy, in general. With increased understanding of gain- and loss-of-function mechanisms of genetic forms of ALS, ASOs therapies have also been tested principally tested in *SOD1* and *C9ORF72* models to target the mutant forms of RNA but not the wild-type.

The first study using an ASO to target *SOD1* showed an effective silencing of *SOD1* and reduced mutated SOD1 protein throughout the brain and spinal cord of *SOD1^G93A^* rats (Smith et al., 2006). Infusion of ASOs complementary to hSOD1 mRNA extended survival in *SOD1^G93A^* rats (Smith et al., 2006). Given these promising preclinical results, the ASO IONIS-SOD1_Rx_ (ISIS 333611 and BIIB067) has been proposed as a therapeutic strategy for *SOD1*-link ALS and has been clinical tested. In a phase I testing, intrathecal administration of the ASO IONIS-SOD1_Rx_ was showed to be both practical and safe in SOD1 ALS patients (Miller et al., 2013). A phase Ib/IIa trial (NCT02623699) is currently underway to further evaluate safety, tolerability, and pharmacokinetics of IONIS-SOD1_Rx_. Altogether, the preclinical and clinical tests suggest that ASOs delivered to the CNS represent a feasible treatment for *SOD1*-related ALS and are safe, however, ASOs are not specific for mutant over wild-type SOD1 and the long-term effects of the reduction of SOD1 need further investigation.

In addition, silencing of *SOD1* can be induced by exon skipping of h*SOD1* using ASOs complementary to splicing regulatory elements on the primary transcript (Biferi et al., 2017). For instance, administrating an exon-2-targeted ASO embedded in a modified U7 small-nuclear RNA and delivered by AAV10, in either newborn or adult (P50) *SOD1*^G93A^ mice, was shown to increase survival and restore neuromuscular function (Biferi et al., 2017). These recent findings provide new hope for treatment of ALS and open perspectives for a clinical development.

Strong evidence supports that the mechanism by which the GGGGCC repeat expansion in C9orf72 causes the diseases is by toxicity of RNAs that they generate. Thus early development of ASO-based therapeutics for C9orf72 ALS focused on reducing gain-of-function toxicity associated with the repeat expansion. Testing of the efficacy of ASO-based therapeutics for C9orf72 was initially performed on clinically relevant human IPSC-derived neurons and fibroblasts (Donnelly et al., 2013; Lagier-Tourenne et al., 2013; Sareen et al., 2013). More recently, ASOs were also evaluated in mouse models expressing the expanded C9orf72 (O’Rourke et al., 2015; Jiang et al., 2016).

Antisense oligonucleotides were designed to bind within the GGGGCC repeat expansion or within surrounding N-terminal regions of the C9orf72 mRNA transcript to either degrade the transcript or block the interaction between the repeat expansion and RNA-binding proteins (Donnelly et al., 2013). ASOs effectively reduced RNA foci formation, dipeptide proteins, increased survival from glutamate excitotoxicity and restored normal gene expression markers (Donnelly et al., 2013; Lagier-Tourenne et al., 2013; Sareen et al., 2013; O’Rourke et al., 2015; Jiang et al., 2016). These promising findings suggest that ASO-based therapy can be a powerful way for treating C9orf72 ALS. They also provided the basis for the initiation of the first C9orf72 ASO clinical trial that is anticipated to start by the end of 2018.

These planned ASOs trials in ALS as well as ongoing trials of ASOs in SMA, Huntington’s disease and Alzheimer’s disease will enhance our understanding of this therapeutic approach. Importantly, positive outcomes from these clinical trials will revolutionize the treatment of genetically mediated neurodegenerative diseases.

### Small Molecules Targeting RNA

RNAs adopt discrete secondary and tertiary structures and have pivotal roles in biology and diseases (Bernat and Disney, 2015). The ALS-associated C9orf72 GGGGCC repeat RNA can stably fold to into a four-stranded structure formed by the stacking of planar tetrads of four guanosine residues, termed G-quadruplex (Huppert, 2008; Fratta et al., 2012). This G-quadruplex structure can affect various RNA processing including splicing and translation (Simone et al., 2015). In particular, the C9or72 repeat RNA G-quadruplexes have been shown to specifically sequester RNA-binding proteins and have toxic functions (Haeusler et al., 2016). GGGGCC repeat RNA sequence can also adopt a hairpin structure in addition to G-quadruplexes (Haeusler et al., 2014; Su et al., 2014). Hairpin is composed of a base-paired stem and a loop and it can affect transcription and alternative splicing (Kuznetsov et al., 2008). Targeting these RNA structures of the C9or72 repeat is a potential therapeutic strategy.

Recent developments in technologies and approaches have made the long sought-after goal of developing small-molecule drugs that target RNA possible (Disney, 2013; Bernat and Disney, 2015; Connelly et al., 2016). Small molecules binding to RNA hairpin or G-quadruplex structure have been identified (Di Antonio et al., 2012; Su et al., 2014). This has provided the springboard to initiate the search for small molecules that can specifically target C9orf72 repeat RNA and hinder pathogenic interactions with RNA-binding proteins and/or by interfering with RAN translation (Su et al., 2014; Simone et al., 2018) (Figure 2C).

Su et al. (2014) showed that (GGGGCC)_8_ RNA can adopt a hairpin structure in equilibrium with a quadruplex structure. They designed three compounds targeting mainly the hairpin structure of the (GGGGCC)_n_ RNA and showed that the bioactive small molecule 1a significantly inhibited RAN translation and foci formation in cultured cells (GGGGCC)_66_ repeat expansion and in patient-derived neurons (Su et al., 2014). However, these small molecules were only tested *in vitro* on cellular models. Recently, a drug screen study to identify compounds that specifically target the *C9orf72* RNA G-quadruplex structure led to the identification of three lead compounds (Simone et al., 2018). These compounds were then functionally validated as ALS therapeutics in *C9orf72* IPSC-derived neurons and *C9orf72* repeat-expressing fruit flies. Interestingly, two of the lead compounds reduced RNA foci formation and the levels of toxic dipeptide repeat proteins in IPSC-derived spinal motor neurons and cortical neurons (Simone et al., 2018). The most effective small molecule (DB1273) was then tested *in vivo* on *C9orf72* repeat-expressing fruit flies and was found to significantly reduce dipeptide repeats levels. Furthermore, D1273 improved the survival of the fruit flies (Simone et al., 2018). These studies support the further development of small molecules that selectively bind GGGGCC RNA as a therapeutic strategy for C9orf72 ALS and FTLD.

## Limitations of RNA-Targeted Therapeutic Strategies

RNA-targeted therapeutic approaches offer a treatment strategy with greater specificity, improved potency, and decreased toxicity compared to the small molecules against traditional drug targets (signaling proteins). They represent an important way to treat ALS and other neurodegenerative diseases that need to be considered in the near future. However, there are still some concerns and challenges to overcome for ALS therapeutic applications.

Off-target effects RNAi and ASO remain an important consideration though thorough toxicological and safety research prior to clinical application can diminish some of this concern. The negative charge of siRNA and ASO as well as their size makes it difficult for them to cross the cell membrane. Viral packing is currently widely used to deliver ASO and siRNA into cells. Although, viral vectors are highly efficient as transfer vehicles, immunogenicity of the viral vectors is a major concern. Various other delivery strategies such as nanoparticles, liposomes and aptamers could be more effective and safe. Efforts are also underway to chemically stabilize siRNA, which will avoid the need for viral vectors (Castanotto and Rossi, 2009). RNA foci and dipeptide products are generated from both sense and antisense directions of the C9orf72 transcript. However, ASOs for C9orf72 ALS preferentially target sense strand transcripts. There may be a need to design ASO strategies to target toxic RNA transcribed from both directions in order to adequately treat the C9orf72 ALS (Schoch and Miller, 2017). Furthermore, ASO-based therapeutic strategy for C9orf72 ALS only target gain-of-function mechanisms, but loss-of-function mechanisms may also act in synergy to cause pathogenesis in C9orf72 ALS. It is very plausible that an integrated therapeutic approach to inhibit toxic RNA foci/dipeptide repeat protein formation and restore normal levels of C9orf72 may be necessary to fully address the cellular deficits in C9orf72 ALS.

## Conclusion

TDP-43, SOD1, FUS, and C9orf72 mutations are involved at various aspects of RNA processing and many of which are shared. It is becoming clear that impaired RNA regulation and processing is a central feature ALS pathogenesis. Given that defects at multiple steps of RNA processing impair cellular function and survival, RNA metabolism can be considered an essential target for therapeutic intervention for ALS and other neurodegenerative disease such as FTLD. The application of RNA-based therapies to modulation of gene and subsequent protein expression is an attractive therapeutic strategy. The preclinical testing of RNA-based therapies targeting SOD1 and C9orf72 mutations are indeed very promising. Similar studies are yet to be undertaken for FUS and TDP-43 mutations. RNA-based therapies could be considered in the future for the treatment of ALS.

## Author Contributions

SP contributed to the idea conception and overall review design. ZB and SP wrote the manuscript.

## Conflict of Interest Statement

The authors declare that the research was conducted in the absence of any commercial or financial relationships that could be construed as a potential conflict of interest.
